# Editorial: From bench to bedside in gastric cancer: diagnosis, prognosis, and treatment, volume II

**DOI:** 10.3389/fmed.2025.1562989

**Published:** 2025-03-11

**Authors:** Avtar Singh Meena, Alberto Bongiovanni

**Affiliations:** ^1^Department of Biotechnology, All India Institute of Medical Sciences (AIIMS), New Delhi, India; ^2^Osteoncology and Rare Tumors Center (CDO-TR), IRCCS Istituto Romagnolo per lo Studio dei Tumori (IRST), “Dino Amadori”, Meldola, Italy

**Keywords:** gastric cancer, COVID-19, pyroptosis (PS), ferroptosis, artificial intelligence

Gastric cancer (GC) is a major health concern worldwide, with high mortality rates, particularly in advanced stages (1). It is a multifactorial disease influenced by genetic, environmental, and lifestyle factors (2–5). Prevalence rates of GC vary across regions, with higher incidence observed in East Asia, Eastern Europe, and parts of Latin America (6, 7). Despite advancements in diagnostic and therapeutic approaches, the prognosis for GC remains poor, largely due to its late-stage diagnosis and the development of drug resistance (8, 9). The disease is often diagnosed in its advanced stages when it has already metastasized, making it difficult to treat effectively. The COVID-19 pandemic has further underscored the vulnerability of individuals with pre-existing conditions, including GC (10–12). Studies indicate potential molecular links between GC and COVID-19, particularly in immune-related pathways (13). Additionally, the interplay between tumor immunity and inflammatory processes, such as pyroptosis, contributes to GC progression and immune evasion (14, 15). Despite these insights, GC remains difficult to cure due to complex molecular mechanisms, tumor heterogeneity, and the lack of effective targeted therapies. Moreover, challenges in early detection and the absence of a reliable biomarker further hinder successful treatment outcomes. The exploration of new therapeutic avenues, including immune modulation and targeting pyroptosis-related genes, offers hope for improving GC management. Despite extensive research on GC biomarkers, patient prognosis remains poor, underscoring the need for less-invasive diagnostic tools with cancer-specific biomarkers. These advancements could improve early detection, recurrence prediction, and chemotherapy customization, thereby enhancing patient care and survival. While surgery remains the primary treatment, successful outcomes rely on accurate pre-operative diagnosis and staging. Identifying novel diagnostic techniques and molecular biomarkers is crucial to improving diagnostic accuracy, prognostic assessments, and enabling personalized treatments.

The COVID-19 pandemic has illuminated the vulnerabilities of individuals with pre-existing conditions, including GC, a major health threat (16–20). Despite research linking GC and COVID-19, the underlying molecular mechanisms remain unclear. Ma et al. conducted a bioinformatics study that revealed 209 shared differentially expressed genes (DEGs) between the two diseases, enriched in immune-related pathways like neutrophil activation and cytokine activity. They identified ten hub genes, including CDK1, KIF20A, and UBE2C, which could serve as therapeutic targets for managing both conditions (Ma et al.). The authors also explored regulatory networks involving transcription factors and microRNAs, proposing ten candidate drugs, such as ciclopirox and dasatinib. This work provides valuable insights into shared mechanisms and therapeutic strategies, urging further research for clinical validation. In parallel, Khamis et al. investigated the role of pyroptosis-related genes in GC, focusing on their potential as therapeutic targets. Pyroptosis, a form of programmed cell death linked to inflammation, plays a key role in tumor immunity and the tumor microenvironment (TME). Their research identified a six-gene pyroptosis signature (IL6, ELANE, GSDME, TIRAP, PYCARD, and CASP3) that could predict GC patient survival (Khamis et al.). They also highlighted immune-related pathway discrepancies between low- and high-risk groups, with the latter showing reduced immune activation, which correlated with worse outcomes. This underscores the importance of exploring pyroptosis and immune interactions in GC treatment, with potential for personalized therapies.

Lipid metabolism is another critical factor in GC development, as tumor cells exploit pathways like PI3K/Akt/mTOR to enhance lipid metabolism, immune evasion, and treatment resistance (21–23). Wang et al. developed a lipid metabolism-related gene (LMRG) signature using TCGA and GEO datasets, identifying five key genes (APOA1, BCHE, CYP19A1, PLA1A, and STARD5) that can predict prognosis. High-risk patients displayed worse clinical features and immunosuppressive TME, while low-risk patients responded better to immunotherapy and chemotherapy (Wang et al.). These findings suggest that lipid metabolism could play a central role in GC progression and response to treatment, offering new biomarkers and therapeutic strategies. Diagnostic capabilities among endoscopists vary significantly, with intermediate-level practitioners often showing lower diagnostic competence than experts. Zhang et al. assessed an AI-assisted diagnostic system (AIAG) using gastroscopy images from challenging cases. The AIAG demonstrated diagnostic performance similar to intermediate endoscopists and enhanced specificity in diagnosing gastric neoplasms. Although promising, the study's single-center, retrospective design and limited dataset call for future multi-center, prospective studies to optimize AI systems for broader clinical application, reinforcing AI's potential in supporting diagnostic accuracy.

Systemic inflammation also affects GC prognosis, with blood-derived markers such as the lymphocyte-to-monocyte ratio (LMR) offering valuable predictive insights (24–27). Mei et al. conducted a meta-analysis involving 815 GC patients treated with immune checkpoint inhibitors (ICIs), finding that a high pre-treatment LMR predicted better overall survival (OS) and progression-free survival (PFS). However, the study's limitations, including regional focus and variable LMR cut-off values, suggest that further research with diverse populations and standardized protocols is necessary. Ferroptosis, a regulated form of cell death, plays a crucial role in cancer therapy, including GC (15, 28–30). Zheng et al. demonstrated that proton pump inhibitors (PPIs) induce ferroptosis in GC cells by upregulating miR-124-3p, which inhibits NRF2 and triggers cancer cell death. These findings open new avenues for GC treatment, although further exploration of other microRNAs and their roles in ferroptosis is needed to develop effective strategies. Lastly, Zhu et al. reviewed the impact of brain metastases on GC patients, which significantly affect survival and quality of life. Despite advances in understanding metastasis, the exact mechanisms driving brain metastasis in GC remain largely unknown. The brain's unique features, such as the blood-brain barrier and its microenvironment, complicate metastasis. Clinical human studies are crucial for deeper understanding, while the development of screening methods and novel therapies, including immune checkpoint inhibitors, offers hope for improved treatment outcomes and enhanced survival for high-risk patients.

The integration of bioinformatics, systems biology, and emerging therapeutic strategies is pivotal for advancing the understanding and treatment of gastric cancer. The shared molecular mechanisms across various conditions, such as COVID-19, pyroptosis, and lipid metabolism, open avenues for novel therapeutic interventions. Additionally, the potential of AI in enhancing diagnostic competence and blood-based markers for prognostic evaluation further contributes to improving patient outcomes. However, future research must focus on validating these findings in larger, more diverse cohorts to refine treatment approaches and optimize diagnostic tools.
